# Cytokines that Modulate the Differentiation of Th17 Cells in Autoimmune Uveitis

**DOI:** 10.1155/2021/6693542

**Published:** 2021-03-16

**Authors:** Kailei Guo, Xiaomin Zhang

**Affiliations:** Tianjin Key Laboratory of Retinal Functions and Diseases, Eye Institute and School of Optometry, Tianjin Medical University Eye Hospital, Tianjin 300384, China

## Abstract

Increasing evidence has suggested that T helper 17 (Th17) cells play a central role in the pathogenesis of ocular immune disease. The association between pathogenic Th17 cells and the development of uveitis has been confirmed in experimental and clinical studies. Several cytokines affect the initiation and stabilization of the differentiation of Th17 cells. Therefore, understanding the mechanism of related cytokines in the differentiation of Th17 cells is important for exploring the pathogenesis and the potential therapeutic targets of uveitis. This article briefly describes the structures, mechanisms, and targeted drugs of cytokines—including interleukin (IL)-6, transforming growth factor-*β*1 (TGF-*β*1), IL-1*β*, IL-23, IL-27, IL-35, IL-2, IL-4, IL-21, and interferon (IFN)-*γ*—which have an important influence on the differentiation of Th17 cells and discusses their potential as therapeutic targets for treating autoimmune uveitis.

## 1. Introduction

**Figure 1:**
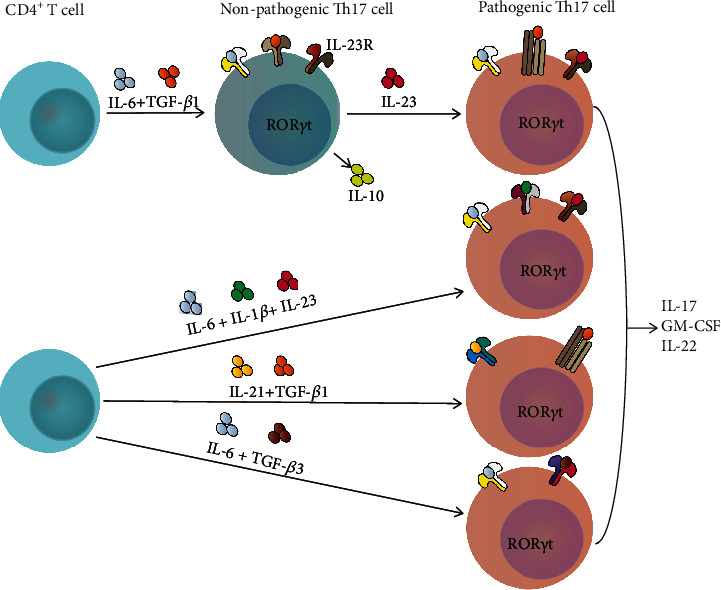
Differentiation of two subsets of Th17 cells in mice.

CD4+ T cells can differentiate into T helper (Th) or T regulatory cell (Treg) subsets. Th cell subsets have been classified into Th1, Th2, Th9, Th17, Th22, and follicular helper T (Tfh) cells based on the secreted cytokines and the special transcription factors [1, 2]. Th17 cells discovered in 2005 can produce the characteristic cytokine interleukin (IL)-17A and the lineage-specific transcription factor retinoid-related orphan receptor gamma t (ROR*γ*t) [3]. Th17 cells are implicated in the pathogenesis of many autoimmune diseases and appear to be divided into two distinct subpopulations *in vivo*, pathogenic and nonpathogenic populations. Pathogenic Th17 cells are generally considered to induce immune cells by secreting proinflammatory cytokines, including IL-17A, IL-17F, IL-22, and granulocyte macrophage-colony stimulating factor (GM-CSF), thus causing tissue damage [4]. Conversely, nonpathogenic Th17 cells do not induce tissue inflammation and may have some function in inhibiting autoimmune inflammation. They can negatively regulate immune responses by producing immune regulatory cytokines, such as IL-10 [5]. Currently, a series of studies have corroborated that naïve CD4+T cells differentiate into pathogenic Th17 cells or nonpathogenic Th17 cells depending on the different cytokines in the environment. It is widely acknowledged that nonpathogenic Th17 cells can be induced by transforming growth factor-*β*1 (TGF-*β*1) combined with IL-6, while pathogenic Th17 cells can be induced by IL-6+TGF-*β*1+IL-23, IL-21+TGF-*β*1, IL-6+TGF-*β*3, or IL-6+IL-1*β*+IL-23 in mice (see Figure 1) [4, 6–8]. Aside from the cytokines mentioned above, IL-27, IL-35, IL-2, IL-4, and interferon (IFN)-*γ* have some distinct effects in suppressing the differentiation of and regulating the pathology of Th17 cells [9].

Autoimmune uveitis is an immune-mediated disease with an unclear etiology and includes Vogt-Koyanagi-Harada disease (VKH), Behcet's disease (BD), sympathetic ophthalmia (SO), birdshot retinochoroidopathy (BSRC), and ocular sarcoidosis [10]. The experimental autoimmune uveitis (EAU) model serves as an animal model of autoimmune uveitis. The EAU model can be divided into antigen-induced EAU (aEAU) models and T cell-induced EAU (tEAU) models, as well as more recently developed spontaneous models. In the aEAU model, mice or rats are immunized with a retinal antigen, such as retinal S-antigen/arrestin (S-Ag) or interphotoreceptor retinoid-binding protein (IRBP) in complete Freund's adjuvant (CFA) [11]. Antigen-specific T cells derived from aEAU animals can induce tEAU by intravenous injection after *in vitro* expansion. Transgenic mice that express a T cell receptor (TCR) specific for IRBP are used in the spontaneous model [12]. EAU models are extremely useful for obtaining insights into the mechanisms that might lead to uveitis in humans.

Initially, Th1 cells were thought to be the major pathogenic mediator of autoimmune uveitis. With the discovery of Th17 cells in the peripheral blood mononuclear cell (PBMC) population of healthy humans and elevated Th17 levels in patients with active uveitis but decreased levels after treatment, Th17 cells gradually came to occupy the same or an even more important position in the development of uveitis [9]. It has been proven that mice with *Stat3* gene knock-out in CD4+T cells cannot produce Th17 cells and cannot lead to the development of EAU [13]. Treatment with IL-6 receptor monoclonal antibody is able to alleviate EAU mostly due to the inhibition of Th17 cell response [14], suggesting the pivotal role of Th17 cells in the EAU progress. Therefore, how to inhibit Th17 cell differentiation or induce pathogenic Th17 cells to transform into nonpathogenic Th17 cells has become a central issue for the treatment of autoimmune uveitis. Here, we focus on the structures and the signaling pathways of the cytokines which regulate Th17 cell differentiation and discuss the potential therapeutic targets for the treatment of autoimmune uveitis.

## 2. Interleukin -6

### 2.1. IL-6 and IL-6 Receptor

The IL-6 family of cytokines is composed of 10 members, including IL-6, IL-11, IL-27, IL-35, IL-39, oncostatin M (OSM), leukaemia inhibitory factor (LIF), ciliary neurotrophic factor (CNTF), cardiotrophin 1 (CT-1), and cardiotrophin-like cytokine factor 1 (CLCF1) [15]. The cytokine IL-6 is a major and multifunctional regulatory agent. The roles of IL-6 in inflammatory and autoimmune diseases have been widely described. Various types of cells are capable of secreting IL-6, including monocytes, T cells, B cells, epithelial cells, adipocytes, and some tumor cells [16].

**Figure 2:**
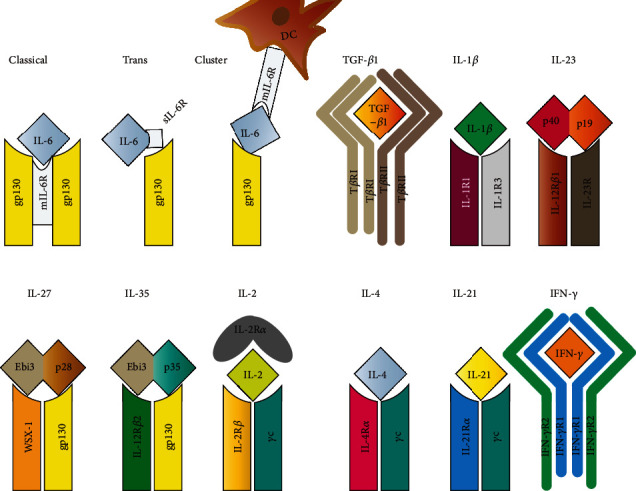
The structures of IL-6, TGF-*β*1, IL-1*β*, IL-23, IL-27, IL-35, IL-2, IL-4, IL-21, IFN-*γ*, and their receptors in CD4+ T cells.

IL-6 receptor (IL-6R) is mainly expressed in T cells, monocytes, activated B cells, and neutrophils [16]. At present, IL-6 signal transmission is achieved through three pathways, classical, trans, and cluster signaling. The three pathways agree in the structure of IL-6R, which is a heterodimer composed of an *α* chain, IL-6R*α*, and a *β* chain, glycoprotein 130 (gp130). Gp130 is the defining subunit of the receptor complexes of all cytokines in the IL-6 family [17]. In the classical pathway, IL-6 binds with a high affinity to the membrane-bound IL-6R (mIL-6R) and transduces signaling via gp130. Meanwhile, IL-6R*α* can be proteolytically shed and bind to IL-6 as a soluble receptor (sIL-6R*α*) and associate with gp130 on target cells in the transsignaling [18]. However, in IL-6 cluster signaling, the IL-6/IL-6R*α* complex forms internally in dendritic cells (DCs) and interacts with gp130 expressed on antigen-specific T cells, a pathway which may be relevant to multiple T lymphocyte-associated autoimmune diseases (see Figure 2) [19]. After IL-6 complexes with IL-6R*α* and gp130, gp130 phosphorylates the Janus kinase family, including JAK1/2 and tyrosine kinase 2 (Tyk2), and then activates the signal transducer and activator of transcription1/3 (STAT1/3), resulting in various biological functions [20].

### 2.2. Promotional Effect of IL-6 on the Differentiation of Pathogenic Th17 Cells

**Figure 3:**
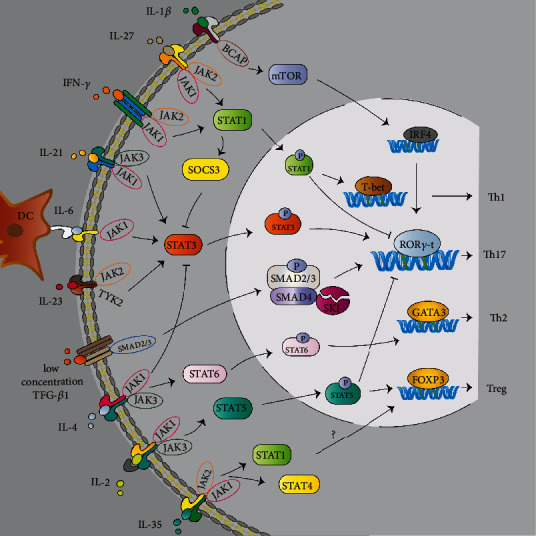
The main pathways of IL-6, TGF-*β*1, IL-1*β*, IL-23, IL-27, IL-35, IL-2, IL-4, IL-21, and IFN-*γ* signaling to induce the differentiation of Th17 cells.

IL-6 contributes to the development of autoimmune diseases by promoting the differentiation and expansion of Th17 cells and suppressing Tregs. As mentioned above, IL-6 is essential for both pathogenic and nonpathogenic differentiation of Th17 cells. IL-6/IL-6R signaling can activate both STAT3 and STAT1 via the JAK family. STAT3 activation up-regulates the expression of ROR*γ*t transcription factor and promotes the differentiation of Th17 cells (see Figure 3) [21]. Heink et al. have reported that the IL-6 cluster signaling transmitting by Sirp*α*+ DCs promotes the differentiation of pathogenic Th17 cells by inducing earlier activation of STAT3 signaling and more robust expression of IL-23R, while the classical IL-6 signaling suppresses the differentiation of Foxp3+ Treg cells in experimental autoimmune encephalomyelitis (EAE) [22]. STAT3 activation also can be induced by IL-21 and IL-23, which will be elaborated upon below. In contrast to STAT3, STAT1 activation inhibits the differentiation of Th17 cells. The ratio of phosphorylated STAT3 (p-STAT3) to p-STAT1 induced by cytokines may predict whether highly proinflammatory Th17 cells will be produced [23]. It has been demonstrated that STAT3 activation is retained while STAT1 activation is suppressed when Th17 cells are stimulated by IL-6 [24].

In addition, IL-6 influences the expression of IL-23R and IL-1R via regulation of microRNAs, such as the microRNA-183-96-182 cluster, which can promote Th17 cell pathogenicity [25]. A recent study reports that the IL-6/STAT3 pathway inhibits the expression of transcription factor regulatory factor X1(RFX1), which binds to the X boxes of MHC class II genes. It has been proven *in vitro* that the deficiency of RFX1 can increase the differentiation of naïve CD4+ T cells into Th17 cells [26]. In conclusion, the IL-6/STAT3 signaling pathway plays a critical role in mediating the differentiation and pathogenicity of Th17 cells.

### 2.3. Therapeutic Potential of Blocking IL-6 in Autoimmune Uveitis

The proinflammatory role of IL-6 in autoimmune uveitis has been widely described. Both IL-6-deficiency and intravitreal injection of anti-IL-6 antibody can effectively attenuate EAU by inhibiting Th17 cell development [27, 28]. The levels of IL-6 are elevated in the serum, plasma, tear, PBMCs, aqueous humor (AqH), and vitreous fluid of patients with active autoimmune uveitis [29–33]. Therefore, treatment targeting the IL-6 and IL-6R has emerged as an innovative therapeutic approach for autoimmune uveitis.

Currently, anti-IL-6 or IL-6R therapy is used worldwide in various autoimmune diseases, such as rheumatoid arthritis (RA), juvenile idiopathic arthritis (JIA), systemic sclerosis, and uveitis [34]. Tocilizumab and Sarilumab are both monoclonal antibody inhibitors of IL-6R, while ALX-0061 is a bispecific nanobody with a high affinity for IL-6R [35]. Sirukumab, Siltuximab, Olokizumab, Clazakizumab, and EBI-031 are biological agents that target IL-6 [36]. Among these, the efficacy of treatment with Tocilizumab has been reported in noninfectious uveitis (NIU), BD, and severe JIA-associated uveitis [37–39], and a clinical trial assessing the efficacy and safety of tocilizumab for treating refractory BD is in progress (ClinicalTrials.gov NCT03554161) (see Table 1). The efficacy of Sarilumab to treat posterior segment NIU has also been reported. In a phase 2 study, 58 patients (one eye per patient) with noninfectious intermediate, posterior, or panuveitis were treated with 200 mg of subcutaneous Sarilumab or placebo every two weeks for 16 weeks. The results demonstrated that patients treated with Sarilumab have a better mean best-corrected visual acuity than placebo patients [40]. The use of the other agents has not been reported in uveitis.

## 3. Transforming Growth Factor-*β*1

### 3.1. TGF-*β*1 and TGF-*β*1 Receptor

The TGF-*β* family regulates a wide variety of cellular processes, such as proliferation, differentiation, migration, and apoptosis. Their effects are context-dependent, depending on the concentration, target cells, and growth stage [41]. The human TGF-*β* family includes three TGF-*β* isoforms (TGF-*β*1, 2, and 3), activins, nodal, bone morphogenetic proteins (BMPs), and growth and differentiation factors (GDFs) [42]. Among these, TGF-*β*1 is ubiquitously expressed in mammalian cells and acts as an essential regulator for immune cell proliferation and differentiation. TGF-*β*1-null mice show an early-onset multifocal inflammation phenotype because of the vital role of TGF-*β*1 in suppressing immune responses [43]. All three TGF-*β* ligands transmit signals through a heteromeric complex of type I and type II TGF-*β* receptors (T*β*RI and T*β*RII). Upon binding of TGF-*β* to T*β*RII, T*β*RI is recruited and phosphorylated. Phosphorylated T*β*RI phosphorylates the downstream mediators, the highly homologous TGF-*β* receptor regulates mothers against decapentaplegic homolog 2 and 3 (SMAD2 and SMAD3), which then combine with SMAD4 and enter the nucleus where they activate or repress the transcription of TGF-*β* target genes [44].

### 3.2. Promotional Effect of TGF-*β*1 at Low Concentrations on the Differentiation of Th17 cells

As a context-dependent cytokine, TGF-*β*1 can promote the expression of IL-23R and ROR*γ*t to induce Th17 cell differentiation when combined with IL-6 or IL-21 at low concentrations. Meanwhile, TGF-*β*1 represses IL-23R expression and supports Foxp3+ Treg cell generation when combined with IL-2 or at high concentrations [7, 8, 45]. Furthermore, a recent publication suggests that IL-6+TGF-*β*3-induced Th17 cells may be more pathogenic than that induced by IL-6+TGF-*β*1 in EAE [46]. The differentiation of Th17 cells is profoundly diminished in mice with TGF-*β*1 deficiency or TGF-*β*1 signal locking but enhanced in TGF-*β*1 transgenic mice [8, 47]. Altogether, these observations strongly confirm that TGF-*β*1 plays a key role in Th17 cell differentiation. However, Ghoreschi et al. have found that TGF-*β*1 is not essential when Th17 cells are induced in IL-6+IL-1*β*+IL-23 conditions *in vivo* [48]. Therefore, further exploration of the more concrete and comprehensive role of TGF-*β*1 on Th17 cells continues.

The canonical TGF-*β* pathway involves SMAD2/3/4, which has been mentioned above. A report supports the idea that SMAD2 and SMAD3 have opposite functions for Th17 cell differentiation when they act as transcription cofactors of STAT3 at different phosphorylation states which are independent of SMAD4 in a collagen-induced arthritis model [49]. Namely, phosphorylated SMAD2 serves as a STAT3 coactivator, while unphosphorylated SMAD3 serves as a STAT3 corepressor in regulating the expression of *Rorc* and *Il17a* gene, respectively. Another report elucidates that SMAD3 also acts as a STAT3 corepressor in regulating the T-lymphoma invasion and metastasis protein (Tiam1) expression in Th17 cells. Tiam1 deficiency reduces the expression of IL-17A partially and slows down the progression of EAE [50]. Therefore, the cross-regulation between SMAD2/3 and STAT3 signaling pathways could balance the interplay between TGF-*β*1 and IL-6 or IL-21 in inducing Th17 cell differentiation.

Currently, the interaction of TGF-*β*1 with SMAD4 in Th17 cell differentiation has been investigated with some results. A series of experiments show that SMAD4 itself does not possess a suppressive or supportive function in Th17 cell differentiation. Otherwise, SMAD4 has been found to interact with SKI, a transcriptional repressor, to suppress Th17 differentiation. SKI promptly suppresses *Rorc* gene expression and Th17 cell differentiation through the mediation of SMAD4. However, TGF-*β*1 could directly induce the degradation of SKI and prevent it from binding with SMAD4, then offset the suppression effect of SKI in Th17 cell differentiation, which is partially SMAD2/3-dependent [51, 52]. In addition to the canonical TGF-*β*/SMAD signaling pathway, TGF-*β*1 can activate some other noncanonical signaling pathways such as the mitogen-activated protein kinase (MAPK) pathway, Rho family GTPases, and nuclear factor-*Κ*B(NF-*Κ*B) pathway, which may also play a role in Th17 cell differentiation [53].

### 3.3. Therapeutic Potential of Blocking TGF-*β*1 in Autoimmune Uveitis

It has been reported that the lymph node cells from mice immunized with IRBP would acquire pathogenicity when stimulated by IL-23+IL-6+TGF-*β*1 and immunizing antigen [54]. In some clinical research, the serum levels of TGF-*β*1 are elevated in active HLA-A29-associated BSRC patients and BD patients [55, 56]. Shimizu et al. have reported that skin lesion-infiltrating CD4+ T cells express stronger staining intensity for TGF-*β*1 in active BD patients than those CD4+ T cells infiltrating into primary erythema nodosum [57]. In contrast, one research group has found that the methylation levels of IL-4 and TGF-*β*1 are significantly upregulated, and the corresponding mRNA expression is down-regulated in active BD patients [58]. The multiple functions of TGF-*β*1 may explain the opposite result in CD4+T cell differentiation, which refers to regulating the balance of Th17 cells versus Tregs in autoimmune uveitis.

Currently, a phase 1 study for Systemic Sclerosis patients with human anti-TGF-*β*1 monoclonal antibody started in 2002 (ClinicalTrials.gov NCT00043706), but no results have been published, and no clinical trials have described the role of TGF-*β*1-related drugs in uveitis. Related animal experimentation reported that rapamycin can decrease Th17 cells but upregulated Tregs in EAU, which may be due to the significant increase in TGF-*β*1 production [59]. The application of TGF-*β*1-related biologic agents in autoimmune uveitis remains to have a long way to go due to the main role of TGF-*β*1 in pathogenic or nonpathogenic Th17 or Treg cell differentiation being relevant to the complex immune environment in various types of autoimmune uveitis and has not been clearly elucidated in previous studies.

## 4. Interleukin-1*β*

### 4.1. IL-1*β* and IL-1*β* Receptor

As a proinflammatory cytokine, IL-1*β* is mainly produced by DCs, monocytes, macrophages, and neutrophils. The cleavage of pro-IL-1*β* in the N-terminal region is facilitated by the active protease caspase-1 to yield the bioactive form. IL-1*β* can activate inflammasomes, recruit inflammatory cells, and enhance T cell activation and TCR antigen recognition. The disorders of IL-1*β* production are related to numerous inflammatory and autoimmune diseases [60]. IL-1*β* is a member of the IL-1 family, which also includes six proinflammatory agonists (IL-1*α*, IL-18, IL- 33, IL-36*α*, IL-36*β*, and IL-36*γ*) and four antagonists (IL-1R antagonist [IL-1Ra], IL-36Ra, IL-37, and IL-38) [61].

IL-1*β* are agonists of the heterodimeric receptor IL-1R which consists of IL-1R1 and IL-1R3. IL-1*β* binds and transmits signals with IL-1R1, while IL-1R3 is an accessory chain [62]. IL-1R is expressed in nearly all tissues and can recruit intracellular adapter molecules, including IL-1R-associated kinase (IRAK), myeloid differentiation factor 88 (MyD88), TNF receptor-associated factor 6 (TRAF6), and B cell adapter for phosphoinositide 3-kinase (BCAP). They activate the downstream pathways, such as the mechanistic target of rapamycin (mTOR), NF-*κ*B, p38, JNK, and MAPK pathways [63, 64].

### 4.2. Promotional Effect of IL-1*β* on the Differentiation of Th17 cells

IL-1*β* signaling plays a significant role in Th17 cell differentiation and the maintenance and proliferation of polarized Th17 cells. It has been confirmed that IL-1*β* synergizes with IL-6 and IL-23 to promote Th17 cell differentiation and that the mechanism involves the transcription factor interleukin regulatory factor 4 (IRF4) [48]. The expression of IRF4 and ROR*γ*t is significantly increased when Th17 cells are induced by IL-6+IL-1*β*, while Th17 cells stimulated with IL-6 only moderately upregulate ROR*γ*t. IRF4-deficient mice are resistant to the induction of Th17 cell differentiation in the EAE model [65]. Sha et al. have reported that the expression of the *IRF4*, *RORC*, *IL17*, *IL21*, *IL22*, and *IL23R* genes in human naive CD4+ T cells which cultured by Th17-polarizing cytokines are inhibited when *IL1R1* gene expression is silenced. Subsequently, they identified that Th17 cell differentiation could also be suppressed when the *IRF4* gene is silenced by siRNA [66]. Therefore, IL-1*β* signaling promotes Th17 cell differentiation mainly via the induction of IRF4. The activation of p38 via TCR may also be required for the induction of IRF4 in Th17 cells [67]. Mailer et al. have reported that IL-1*β* promotes Th17 cell differentiation by inducing the excision of *FOXP3* exon 7 in Crohn's disease [68]. In addition, IL-1*β* and IL-23 drive naive T cells to promote glucose uptake and increase glycolysis in the absence of the costimulatory molecule CD28, which is necessary for Th17 cell differentiation and expansion [69]. BCAP, the intracellular adapter molecule of IL-1R, is critical for IL-1*β*-induced phosphoinositide 3-kinase (PI3K)-AKT-mTOR activation. The deficiency of BCAP and the inhibition of mTOR together completely prevent pathogenic Th17 cell differentiation in the presence of IL-1*β* [64].

IL-1*β* also plays a critical role in the proliferation and survival of polarized Th17 cells. The downstream pathway involves the activation of the I*κ*B kinase (IKKi)-glycogen synthase kinase 3*α*- (GSK3*α*-) mediated AKT-mTOR pathway, which is essential for the regulation of immune responses and cell metabolism [70]. Before IL-1*β* stimulation, AKT forms a complex with GSK3*α* and IKKi, and GSK3*α* serves a function in negatively regulating AKT activation; meanwhile, IKKi is activated and inhibits the function of GSK3*α* after IL-1*β* stimulation, resulting in AKT-mTOR activation and the proliferation/survival of polarized Th17 cells [71].

### 4.3. Therapeutic Potential of Blocking IL-1*β* in Autoimmune Uveitis

The pro-inflammatory role of IL-1*β* in the EAU model has been confirmed. IL-1R-deficient mice are related to a lower number of pathogenic Th17 cells in the retina and an abirritant EAU [60]. The levels of IL-1*β* in serum, tears, and AqH are increased in patients with active HLA-B27-associated uveitis and BD compared with healthy controls [72, 73].

Currently, the biologic agents targeting IL-1*β* mainly include anakinra, canakinumab, gevokizumab, and rilonacept, which have an efficient role in the treatment of uveitis, especially in BD [74]. Anakinra is a recombinant form of human IL-1R antagonist, which blocks the signal transduction of both IL-1*α* and IL-1*β* and has been approved to treat active RA and cryopyrin-associated periodic syndromes (CAPS). Canakinumab and gevokizumab both are monoclonal antibodies to IL-1*β*. Canakinumab has been approved for the treatment of CAPS and systemic juvenile idiopathic arthritis. Rilonacept is an IL-1R fusion protein consisting of the Fc portion of human IgG1 and the human IL-1R. It has also been approved for treating CAPS, but no literature has reported its role in uveitis until now [75]. In BD patients, some studies have found that anakinra and canakinumab are effective in the management of BD-related uveitis, especially in those patients with a long-lasting disease [76, 77]. In addition, gevokizumab was reported to have the ability to rapidly control acute ocular exacerbations in BD patients in a phase 2 study [78]. Moreover, a series of clinical trials have been reported on the safety and efficacy of gevokizumab for the treatment of BD uveitis or NIU (Clinicaltrials.gov NCT01684345, NCT01747538, NCT01965145). However, the randomized, double-masked, placebo-controlled clinical trial of gevokizumab in BD uveitis failed to significantly reduce the time of the first acute ocular exacerbation, and the other two trials designed to evaluate the long-term safety data of gevokizumab in uveitis were cancelled as the Behcet uveitis trial did not meet the primary outcome, although gevokizumab was benefit to preserve visual acuity, reduce the emergence of macular edema, and was well tolerated [79] (Clinicaltrials.gov NCT01965145, NCT02375685, NCT02258854). Overall, controlling the IL-1*β* pathway in uveitis patients deserves further exploration.

## 5. Interleukin-23

### 5.1. IL-23 and IL-23 Receptor

IL-23 was found by Oppmann in 2000 and is a member of the IL-12 cytokine family, which also includes IL-12, IL-27, IL-35, and IL-39 [80]. The heterodimeric cytokines of the IL-12 family consist of an *α* chain (p19, p28, or p35) and a *β* chain (p40 or Epstein-Barr virus-induced gene 3 (Ebi3)). Ebi3 and IL-27p28 form IL-27. Ebi3 also associates with IL-12p35 or IL-23p19 to form IL-35 or IL-39, whereas IL-12p35 and IL-12p40 form IL-12. Similarly, IL-23p19 and IL-12p40 constitute IL-23 [81]. IL-23 is secreted by activated DCs, phagocytic cells, B cells, and dermal Langerhans cells. Multiple lines of evidence have proven that IL-23 plays an important pro-inflammatory role in autoimmune diseases and is critical for the conversion of naïve T cells to homeostatic and pathogenic Th17 effector cells [82].

IL-23 binds to a receptor composed of a unique IL-23R subunit and a *β*1 subunit of IL-12 (IL-12R*β*1) which is shared with IL-12 [83]. IL-23R is expressed on Th17 cells, *γδ* T cells, natural killer (NK) cells, and DCs [82]. Bloch et al. have found that the biological activity of IL-23 is mediated by the interaction of the IL-23p19 subunit with the N-terminal immunoglobulin (Ig) domain of IL-23R, leading to the receptor-mediated restraint of the IL-12p40 subunit to enable a high-affinity interaction with IL-12R*β*1 [84]. IL-23/IL-23 receptor signaling activates STAT3/4 through JAK2/ TYK2.

### 5.2. Promotional Effect of IL-23 on the Differentiation of Pathogenic Th17 Cells

IL-23 is not necessary for the initial stage of Th17 cell differentiation due to the lack of IL-23R on naïve CD4+ T cells [85], but the expression of IL-23R and its exposure to IL-23 in the later stages are vital for evoking the pathogenic potential in Th17 cells [4]. Some cytokines have been discovered that play a key role in upregulating IL-23R expression when naïve CD4+ T cells are exposed to them, such as IL-6, TGF-*β*1, IL-21, and IL-12. When IL-6 is used to stimulate naïve CD4+ T cells, it can upregulate IL-23R expression via the binding of STAT3 to the *IL23R* locus. The activation of STAT3 further increases the miR-183-96-182 cluster, which restrains the activity of forkhead box O1 (FOXO1), a negative transcription factor of pathogenic Th17 cell responses, thus positively regulating pathogenic Th17 cell function [25, 86]. Another study has found that STAT3 increases miR-223-3p to repress the expression of FOXO3, positively regulating IL-23R expression, and thus increasing the pathogenic Th17 cells in the EAU model [87]. IL-21, IL-12, and low concentrations of TGF-*β*1 are also important for the induction of IL-23R. IL-21R deficiency limits IL-23R expression and Th17 cell development in the transgenic EAE model, and the level of *Il23r* mRNA is significantly increased when naive CD4+ T cells are within anti-CD3/CD28 and IL-12 conditions [88, 89]. Some reports suggest that estrogen receptor *α* (ER*α*) signaling increases IL-17A production in Th17 cells by upregulating IL-23R expression in a Let-7f-dependent manner, and this might be a reason for the increasing prevalence of systemic lupus erythematosus (SLE) and multiple sclerosis in women [90]. Whether this mechanism contributes to the increased susceptibility to uveitis in certain women has not been studied.

Currently, it is widely accepted that after the upregulation of IL-23R, IL-23 signaling activates STAT3 through JAK2/ TYK2 and then mediates the transactivation of ROR*γ*t to induce the differentiation of pathogenic Th17 cells and the production of proinflammation cytokines, including IL-17A, IL-17F, IL-22, GM-CSF, and TNF-*α*, thereby driving inflammatory and autoimmune diseases [91]. Furthermore, IL-23 signaling also activates STAT4, which is mainly involved in the IL-12 signaling pathway and has a major effect on the induction of IFN-*γ* in Th1 and Th17 cells [21, 89]. Maturation protein-1 (Blimp-1), induced by B lymphocytes, is also a key IL-23-dependent transcription regulator, and it synergizes with ROR*γ*t to activate Th17 cell-specific inflammatory genes [92]. It should be highlighted that IL-23 acts on not only Th17 cells but also CD8+ T cells, *γδ* T cells, and innate lymphoid cells (ILCs) to induce IL-17 production, and all these cells are involved in mediating autoimmune tissue inflammation [93].

### 5.3. Therapeutic Potential of Blocking IL-23 in Autoimmune Uveitis

It has been confirmed that IL-23 is necessary for the pathogenesis of EAU; IL-23p19 or IL-12p40 subunit-deficient mice show resistance to EAU [94]. In clinical studies, high IL-23 levels have been observed in the serum and PBMCs of patients with active autoimmune uveitis, such as VKH, BD, and BSRC [56, 95, 96]. Human genome-wide association studies (GWASs) demonstrate that several single-nucleotide polymorphisms (SNPs) in the IL-23R genes are linked to the progression of some immune disorders [97], including uveitis. Jiang et al. have identified that the SNPs of IL-23R, including rs17375018 GG and rs11209032 AA, are strongly associated with uveitis [98].

To date, the biological agents that target IL-23 include apilimod mesylate, brazikumab, briakinumab, guselkumab, mirikizumab, risankizumab, tildrakizumab, and ustekinumab [99]. Among these, ustekinumab and guselkumab have been reported in uveitis-related cases. Ustekinumab, which targets the p40 subunit of IL-23 and IL-12, has been reported in three clinical registration studies. A phase 2 study for 16 active BD patients was completed in May 2019 (Clinicaltrials.gov NCT02648581), but no results were published. A phase 2 study for the use of ustekinumab in 11 participants with active intermediate uveitis, posterior uveitis, or panuveitis is expected to be completed in December 2020 (ClinicalTrials.gov NCT02911116). Another phase 2 study for treating 29 patients with noninfectious severe uveitis (NISU) is expected to be completed by January 2022 (Clinicaltrials.gov NCT03847272). Ustekinumab also has been reported as an effective agent for treating BD-related oral ulcers when resisting treatment with colchicine [100]. Therefore, ustekinumab may have positive therapeutic effects on uveitis. Guselkumab inhibits the intracellular and downstream signaling of IL-23 by binding to the p19 subunit [101]. However, a case report described a patient whose sarcoidosis-related panuveitis worsened after receiving guselkumab [102]. Nevertheless, inhibiting IL-23/IL-23R signaling is a promising potential strategy for treating autoimmune uveitis.

## 6. Interleukin-27

### 6.1. IL-27 and IL-27 Receptor

IL-27, which consists of Ebi3 and IL-27p28, was first identified in 2002. Recent advances reveal that IL-27 not only has significant functions in anti-inflammation and immune regulation but also plays an important role in regulating the differentiation and immune response of CD4+ T cells [103]. IL-27 is mainly produced by antigen-presenting cells (APCs) which include DCs, monocytes, and macrophages. IL-27 receptor (IL-27R) is expressed on T lymphocytes, NK cells, mast cells, endothelial cells, and APCs. It is a heterodimer composed of an *α* chain, an orphan cytokine receptor WSX-1, and a *β* chain gp130. IL-27 binds with a high affinity to WSX-1 and transduces signaling via gp130. Indeed, IL-27 belongs to both the IL-6 and IL-12 families due to the structure itself and that of IL-27R. After IL-27 binds to its receptor complexes, gp130 activates the JAK1/2-STAT1 pathways, resulting in anti-inflammatory biological functions [104].

### 6.2. Inhibitory Effect of IL-27 on the Differentiation of Th17 Cells

IL-27-induced STAT1 signaling has been demonstrated to play a role in inhibiting Th17 cell differentiation but activating the differentiation of Th1 cells. Lee et al. have found that STAT1-deficient mice produce reduced amounts of IL-27 and develop more severe EAU [105]. The relevant underlying mechanism includes the contribution of IL-27 mediated-STAT1 phosphorylation to the activation of the T-box transcription factor (T-bet), a specific transcription factor of Th1 cells, which interacts with the Runt-related transcription factor 1 (Runx1) and blocks Runx1-mediated transactivation of ROR*γ*t, thus inhibiting the differentiation of Th17 cells [106]. Another study has found that IL-27-primed naive CD4+ T cells upregulate the expression of programmed death-ligand 1 (PD-L1) in a STAT1-dependent manner, leading to the inhibition of Th17 cell differentiation. The PD-L1 restraint mouse model could partly overcome the defect in the differentiation of Th17 cells [107]. Photoreceptors express IL-27R and respond to IL-27 signaling by producing IL-10 and the suppressor of cytokine signaling 1 (SOCS1) through STAT1-dependent mechanisms [105].

SOCS proteins belong to a family of cytoplasmic proteins that function as negative-feedback regulators of the JAK/STAT pathway and the cytokine signaling. SOCS proteins directly interact with cytokine receptors and/or JAKs to prevent the recruitment of STATs to the signaling complex [108]. SOCS1 and SOCS3 are the best-characterized members of this family. SOCS1 plays a potential role in mitigating ocular inflammation; rats and mice with targeted overexpression of SOCS1 in the retina are partially protected from EAU [109]. A study has found that SOCS3 and IL-27 are temporally correlated with the progression of EAU, and IL-27 may negatively regulate IL-6- or IL-23-induced expansion of Th17 cells through SOSC3-dependent mechanisms [9]. In conclusion, IL-27 and STAT1 are potential biological agents to help prevent autoimmune uveitis.

### 6.3. Therapeutic Potential of IL-27 in Autoimmune Uveitis

Many reports show the inhibitory effect of IL-27 on the differentiation of Th17 cells and the considerable role of IL-27 in suppressing EAU. In clinical research studies, it has been proven that the expression of *IL-27p28* mRNA by PBMCs and the IL-27 expression in serum and supernatants of PBMCs are markedly lower in patients with active VKH and BD compared with healthy subjects, while the *Ebi3* mRNA expression is no different among the groups tested [110, 111].

The effects of both Ebi3 and IL-27p28 on the pathogenicity of Th17 cells in EAU have been studied separately. Stumhofer et al. have reported that IL-27p28 functions as a natural antagonist of gp130-mediated signaling and finally results in the mitigation of cytokine-mediated inflammatory diseases [112]. The overexpression of IL-27p28 in mice contributes to the attenuation of uveitis and the inhibition of the differentiation of Th17 cells, of which the latter is partly attributable to the repression of STAT3 phosphorylation [113]. In Ebi3^−^/^−^ mice immunized with human IRBP to induce EAU, Ebi3 may act as a positive regulator of Th1 cells in the early phase of EAU progress but as a negative regulator of both Th1 and Th17 cells in the late phase of EAU progress [114]. Therefore, in view of the inhibiting effect on Th17 cell differentiation, Ebi3 and IL27p28 deserve further research and may become potential therapeutic targets of uveitis.

Considering the effect of the IL-27 subunit alone on uveitis, studies that explore the effects of recombinant cytokine subunits in the treatment of uveitis are also being carried out [115]. Some reports underscored the promotion of inflammatory diseases by IL-12 and IL-23 (shared p40) and the inhibition of autoimmune diseases, such as uveitis and multiple sclerosis, by IL-27 and IL-35 (shared Ebi3). It has been envisaged that the pairing of an *α* subunit protein with IL-12p40 might promote proinflammatory responses, while coupling with Ebi3 might be associated with immune suppression. However, the results do not follow this prediction. It was discovered that the recombinant IL-27p28/IL-12p40 heterodimeric cytokine treatment outperforms the treatment with p28 alone, which not only inhibits uveitis by inhibiting Th1 and Th17 responses but also promotes the Foxp3 expression and IL-10 production by Treg cells [116]. IL-39, a novel cytokine of the IL-12 family formed by the pairing of Ebi3 and IL-23p19, mediates proinflammatory response in Lupus-like mice but appears to contribute to wound healing by inhibiting inflammatory responses when produced by keratinocytes [117, 118]. Therefore, the combination study of different subunits of the cytokines of the IL-12 family is complex, and whether it will show unexpected results for inflammatory and autoimmune diseases is unknown. Future research should consider not only the advantages but also the risk of potential deleterious consequences.

## 7. Interleukin-35

### 7.1. IL-35 and IL-35 Receptor

IL-35, which consists of Ebi3 and IL-12p35, was discovered by Niedbala and Collison in 2007 [119]. IL-35 is a regulatory cytokine released mainly by CD4^+^Foxp3^+^Treg and regulatory B (Breg) cells [120, 121]. IL-35 receptors use three possible receptor pairs of the *β*2 subunit of IL-12 (IL-12R*β*2) and gp130, including IL-12R*β*2/gp130, IL-12R*β*2/IL-12R*β*2, and gp130/gp130, for signal transduction [122].

### 7.2. Inhibitory Effect of IL-35 on the Differentiation of Th17 Cells

After IL-35 binds to its receptor, the Breg and Treg cells are promoted, but the Th17 and Th1 cells are inhibited. This may be associated with the activation of STAT1 or STAT4 through JAK1/2, thus inhibiting inflammation and reducing the severity of autoimmune diseases [15, 122]. However, the specific mechanism of the regulation of inflammatory and autoimmune diseases by IL-35 remains unknown, and hence, must be studied.

### 7.3. Therapeutic Potential of IL-35 in Autoimmune Uveitis

Recent findings have indicated the protective effect of IL-35 on EAE and EAU [121, 123]. Wang et al. have used genetic engineering to produce highly purified murine rIL-35 and proved that the treatment of EAU in mice with rIL-35 could inhibit uveitis and protect the eyes from pathological effects by inhibiting Th17 and Th1 cells and inducing the expansion of Breg and Treg cells [121]. However, isolating or producing an ample amount of functional IL-35 is challenging and very labor-intensive [124]. Obtaining functional IL-35 more efficiently is still a problem that needs to be solved in the future.

Another study prepared mouse rIL-12p35 and rEbi3 to examine whether the IL-35 subunit proteins showed endogenous immune-suppressive activities, independent of their heterodimeric partner. It was concluded that IL-12p35 could antagonize the pathogenic Th17 cell response and induce the expansion of IL-10- and IL-35-expressing B cells and, thus, ameliorate autoimmune uveitis in mice. It is stated that IL-12p35 shows at least some of the immunomodulatory properties of IL-35, which control autoimmune diseases that affect the neuroretina. Compared to rIL-12p35, rEbi3 shows a less potent effect on the expression of IL-10, IL-12p35, and Ebi3 [125]. IL-12p35 also mediates the amplification of Treg and Breg cells to improve EAE [126]. These results suggested that IL-12p35 might serve as a novel biological agent for the treatment of autoimmune diseases of the central nervous system.

## 8. Interleukin-2

### 8.1. IL-2 and IL-2 Receptor

IL-2 was discovered as an important T cell growth factor that supports the proliferation and generation of T cells, and it is predominantly produced by activated T lymphocytes [127, 128]. Currently, IL-2 plays a crucial role in not only T cell proliferation but also in CD4+ T cell differentiation; it is essential for the differentiation of Treg, Th1, and Th2 cells and for the generation of Th9 cells [129, 130]. However, it inhibits the differentiation of Th17 and Tfh cells and promotes the proliferative expansion of Th17 cells after differentiation [9].

IL-2 receptor (IL-2R) is formed by various combinations of three distinct subunits, including IL-2R*α* (also known as CD25), IL-2R*β* (CD122), and IL-2R*γ* (CD132). IL-2R*γ* is a common cytokine receptor chain known as the *γ* chain (*γ*c) and is shared with the receptors of IL-4, IL-7, IL-9, IL-15, and IL-21 [131]. IL-2R is expressed with different affinities on T cells, B cells, and NK cells, and it is formed by IL-2R*α*/*β*/*γ* in Th17 cells [132]. JAK1 and JAK3 transmit downstream signals composed of a group of IL-2 family cytokines, resulting in the phosphorylation of STAT1, STAT3, STAT4, STAT5, and STAT6 [133].

### 8.2. The Paradoxical Effect of IL-2 on the Proliferation and Differentiation of Th17 Cells

IL-2 is a repressor for the differentiation of Th17 cells, and various mechanisms have been proposed to account for this. IL-2-mediated activation of STAT5 through JAK1/3 inhibits ROR*γ*t expression [134]. STAT5 not only has a negative effect on Th17 cell differentiation but also is essential for Treg development. STAT5 can compete with STAT3 to repress IL-17a transcription and regulate the Th17/Treg balance [135]. Moreover, IL-2 inhibits the expression of mIL-6R*α* and gp130 to inhibit the differentiation of Th17 cells [130]. Currently, the phosphatase and tensin homologue (PTEN), a tumor suppressor, have been found to drive the differentiation of Th17 cells by preventing IL-2 production [136].

Interestingly, IL-2 inhibits the differentiation of Th17 cells but promotes their expansion. This may be a pathogenic mechanism of uveitis and scleritis [9]. Yu et al. have found that Th17 cells produce low levels of IL-2 in EAU and healthy humans, and these low levels of IL-2 are sufficient to promote the persistent expansion of Th17 cells but could not initiate activation-induced cell death, leading to chronic inflammation [137].

### 8.3. Therapeutic Potential of Targeting IL-2 in Autoimmune Uveitis

IL-2 may mediate the development of uveitis by stimulating the proliferation of Th17 cells and the differentiation of Th1 cells [9, 138]. It has been reported that IL-2 and retinoic acid (RA) can promote the induction of antigen-specific type 1 Treg (Tr1) cells in EAU, suggesting that IL-2 might be a promising agent for the treatment of uveitis [139]. The levels of IL-2 are significantly higher in the serum and AqH of active BD patients [31, 140, 141]. The levels of soluble IL-2R in serum are also elevated in patients with sarcoidosis-associated uveitis and HLA-B27-associated uveitis [142].

Daclizumab, a humanized anti-IL-2R*α* drug, has been reported for treating BSRC, BD, and JIA-associated active anterior uveitis [143–146]. However, daclizumab was withdrawn from the market worldwide in 2018 due to unexpected severe adverse events. It has been reported that during long-term daclizumab therapy for 39 patients with refractory posterior uveitis, visual acuity improved in seven patients (18.4%) and worsened in six patients (15.8%). It was especially unfortunate that four patients (10.3%) developed solid tumor malignancies during the 11-year period [147].

Recently, due to the important role of IL-2 in promoting the differentiation of Treg cells and inhibiting the differentiation of Th17 cells, there is growing interest in using IL-2 for the treatment of autoimmune diseases. The trial of low-dose IL-2 treatment has reported positive results in patients with primary Sjögren's syndrome [148]. Klatzmann describes IL-2 as “the corticosteroid of the 21st century” because low-dose IL-2 is well-tolerated in patients with 11 types of different autoimmune diseases (including BD) (Clinicaltrials.gov NCT01988506 and NCT04065672) [149]. Based on current results, low-dose IL-2, rather than anti-IL-2 agents, shows great promise for the treatment of autoimmune diseases.

## 9. Interleukin-4

### 9.1. IL-4 and IL-4 Receptor

IL-4 is a key anti-inflammatory cytokine driving the differentiation of Th2 cells from naïve CD4+T cells, mediating immunoglobulin E (IgE) class switching in B cells, and inducing alternative macrophage activation [150]. IL-4 is mainly produced by mast cells and matured lymphoid cells, such as Th2 cells, NK T cells, basophils, and type II innate lymphoid cells [151].

The IL-4 receptor (IL-4R) has two types. Type I IL-4R is formed by an IL-4R*α* chain (the binding receptor chain for IL-4) and *γ*c, which shares receptors with the IL-2 family and expresses on the surface of lymphocytes and myeloid cells. Type II IL-4R is formed by the IL-4R*α* chain and the IL-13R*α*1 chain, which shares with the IL-13 receptor and expresses on the surface of nonhematopoietic cells and myeloid cells [152]. After IL-4 binds to type I IL-4R in lymphocytes, IL-4R*α* associates with JAK1, and *γ*c associates with JAK3 to activate downstream transcription factor STAT6 [153].

### 9.2. Inhibitory Effect of IL-4 on the Differentiation of Pathogenic Th17 Cells

IL-4 is an essential instructive signal that preferentially promotes the Th2 cell-mediated immune responses, which relies on the activation of JAK1/3-STAT6 and results in the expression of GATA3, a key Th2 cell-specific transcription factor [154]. It works as the negative regulator of Th1 and Th17 cell differentiation. It has been proven that GATA3 suppresses Th17 cell differentiation from naïve CD4+ T cells by downregulating STAT3, STAT4, and ROR*γ*t expression [155]. IL-4 also inhibits the pathogenesis of preexisting or memory Th17 cells by repressing the expression of IL-17A, IL-17F, IL-23R, and ROR*γ*t, which depend on the activation of STAT6 but not GATA3. But the precise mechanisms remain to be determined. However, Th17 cells become resistant to the suppression of IL-4 when being repeatedly stimulated, which may due to the loss of phosphorylating STAT6 capacity for IL-4R [156]. Therefore, the treatment of autoimmune disease using IL-4 may not achieve the desired effect when used repeatedly.

### 9.3. Therapeutic Potential of IL-4 in Autoimmune Uveitis

Although IL-4 can mediate protection by directly promoting Th2 cell response and suppressing Th1 and Th17 differentiation, it also promotes the production of IgE from B cells, which likely mediate autoimmune disease, at least in part [157]. The specific functions of IL-4 in uveitis remain to be explored. Previous research has established that rIL-4 aggravates EAU in rats immunized with S-Ag but decreases the development of uveitis in rats immunized with 60 kDa heat shock protein (HSP60) peptide 336–351. HSPs are a group of intracellular proteins that have a special role in the etiology of BD [158]. The expression of IL-4 is increased when EAU is induced by IRBP peptide 1-20 in CFA. Treating EAU mice with rapamycin or suppressing the reactive oxygen response both reduce the levels of IL-4 and other cytokines related to Th1 and Th17 cells, thus ameliorating EAU [59, 159]. Data from a clinical study suggests that the levels of IL-4 increase in serum and AqH of patients with endogenous uveitis and BD, but the levels are relatively low in BD [160]. However, in some other reports, no significant differences were found between uveitis and healthy control groups for AqH and serum IL-4 levels [161, 162]. Thus, the role of IL-4 in autoimmune uveitis is still unclear, and no IL-4-related biological agents have been developed, although we often think of IL-4 as an anti-inflammatory cytokine.

## 10. Interleukin-21

### 10.1. IL-21 and IL-21 Receptor

IL-21, a pleiotropic cytokine identified in 2000 [163], plays a significant role in promoting CD4+ T cell differentiation and proliferation, effector CD8+ T cell amplification, NK cell activation, B cell proliferation, and Ig production, but inhibits the differentiation, generation, and survival of Treg cells [164, 165]. It is homologous to the IL-2 cytokine family and is predominantly produced by Th17 cells, Tfh cells, and NK T cells [166].

IL-21 receptor (IL-21R) is composed of an *α* chain (IL-21R*α*) and a *γ*c. IL-21R is expressed in various immune cells (T cells, B cells, NK cells, DCs, and macrophages), thyroid cells, and synovial fibroblasts [166]. IL-21/IL-21R signaling has the potential to activate JAK1/3 and subsequently activates STAT3, as well as STAT1 and STAT5 to a lesser extent [167].

### 10.2. Promotional Effect of Blocking IL-21 on the Differentiation of Pathogenic Th17 Cells

IL-21 is not only an autocrine cytokine generated by Th17 cells but also plays an indispensable role in the induction and amplification of Th17 cells. IL-21/IL-21R signaling interacts with JAK1/3-STAT3, inducing the increase of IL-23R expression and the upregulation of ROR*γ*t but inhibiting the expression of Foxp3 [168].

When naive CD4+ T cells are stimulated by IL-6 and TGF-*β*1, IL-6 can induce the secretion of IL-21, which acts through a regenerative feedback mechanism for Th17 cell amplification and differentiation [169]. IL-21- or IL-21R-deficient CD4+ T cells fail to differentiate into Th17 cells in IL-6+TGF-*β*1 conditions, but naive IL-6^−/−^ CD4+ T cells can differentiate into Th17 cells in IL-21 +TGF-*β*1 conditions [7]. In other words, the combination of TGF-*β*1 and IL-21 has the capability to induce mice and human naive CD4+ T cells differentiation into Th17 cells. But IL-21-TGF-*β*1-induced Th17 cells may have different pathogenicity in different species. Mice Th17 cells induced by TGF-*β*1 and IL-21 secrete IL-17A, IL-17F, and IL-22, while human Th17 cells induced by TGF-*β*1 and IL-21 only secrete IL-17A without IFN-*γ* and IL-10. Interestingly, IL-21 increases the level of *IL22* mRNA in human naive CD4+ T cells when given alone, but TGF-*β*1 suppresses the expression of *IL21* and *IL22* mRNA [6]. However, there are no definitive follow-up study reports on whether the human Th17 cells are pathogenic under IL-21+TGF-*β*1 culture conditions, and the specific mechanism of the difference between mice and human Th 17 cells is unclear.

Recently, a series of experiments show that SMAD4, in cooperation with SKI, regulates the IL-21-TGF-*β*1-induced differentiation of Th17 cells by modulating the expression of *Rorc* mRNA. In the absence of TGF-*β*1, SMAD4 cooperates with SKI to repress Rorc transcription to prevent IL-21-induced Th17 cell differentiation. While in the presence of TGF-*β*1, SMAD4 losses its suppression effect due to the TGF-*β*-directed degradation of SKI [53, 170]. Furthermore, it has been confirmed that the CD4+T cells from SMAD4 and T*β*RII double KO mice have the power to differentiate into pathogenic Th17 cells with IL-21 alone. At the same time, activin, a member of the TGF-*β* superfamily, can also interact with IL-21 to induce Th17 cell differentiation by inhibiting SKI [170]. In addition, the production of IL-21 and IL-17 from Th17 cells can be drastically down-regulated by inhibiting Rho-associated kinase 2 (ROCK2). ROCK2 can interact with pSTAT3 in the Th17 cell cytoplasm, which is followed by the recruitment of the ROCK2-STAT3 complex to the Th17 cell-related gene promoters in the nucleus and the production of proinflammatory responses [171].

### 10.3. Therapeutic Potential of Blocking Targeting IL-21 in Autoimmune Uveitis

IL-21 has been shown to play a vital role in the EAU model. The expression of *IL21* and *IL21R* mRNA is significantly increased in the Th17 cells of draining lymph nodes and the spleen in the EAU model compared with normal controls and mice in the recovery phase [172]. In clinical research, IL-21 also has been found to mediate the innate or acquired immune responses in ocular inflammatory and autoimmune diseases, such as primary Sjögren's syndrome, Graves' disease, BD, and age-related macular degeneration [173–177]. The levels of IL-21 in the serum and PBMCs are significantly increased in patients with chronic or recurrent active VKH and active BD [178]. Serum IL-21 is upregulated in patients with active BSRC [56]. Contrastingly, blocking IL-21 restores the homeostasis of T cells in patients with BD [177, 179]. Although there is a distinct relationship between IL-21 and autoimmune uveitis, the biological agents that target IL-21 or IL-21R have not been tested in uveitis. Some animal experiments and clinical trials have discovered that IL-21R-Fc fusion proteins or anti-IL-21 antibodies could be promising drugs to treat SLE and RA [180, 181]. The effectiveness of anti-IL-21/IL-21R or downstream signals in uveitis may be confirmed in future studies.

## 11. Interferon-*γ*

### 11.1. IFN-*γ* and IFN-*γ* Receptor

IFN-*γ* participates in regulating multiple immune processes such as the activation of macrophages, antigen processing and presentation, B cell proliferation and antibody class switching, the production of CD4+ T cells, and CD8+ T cell proliferation [182]. IFN-*γ* is mainly produced by CD4+ and CD8+ effector T cells, NK cells, NK T cells, and *γδ*-T cells [183].

IFN-*γ* receptor (IFN-*γ*R) is formed from the interaction of IFN-*γ*R1 subunits with IFN-*γ*R2 and is expressed on nearly every cell type. IFN-*γ*R1 associates with JAK1 and IFN-*γ*R2 associates with JAK2 to activate downstream transcription factor STAT1 [184].

### 11.2. Inhibitory Effects of IFN-*γ* on the Differentiation of Th17 Cells

IFN-*γ* is the hallmark Th1 cytokine and activates the JAK1/2-STAT1 and the downstream transcriptional target T-bet, a special transcription factor of Th1 cells, resulting in the production of the Th1 phenotype [185]. IFN-*γ* inhibits the differentiation of Th17 cells by activating STAT1 and increasing the expression of SOCS3. SOCS3 has the function to repress the expression of STAT3, resulting in the inhibition of ROR*γ*t and Th17 cell differentiation [186]. IFN-*γ* also suppresses the differentiation of Th17 cells by reducing the expression of IL-23R and TGF-*β*1 [47], but the specific mechanisms are not clear. In addition, the IFN-*γ*-IL-27 axis plays a role in inhibiting Th17 cell differentiation. IFN-*γ* can upregulate the expression of IL-27 in the retinal ganglion and photoreceptor cells to inhibit the differentiation of Th17 cells [9], and it has been confirmed that the IFN-*γ*-producing NK cells can interact with DCs to produce IL-27 in EAU [187].

In addition, IFN-*γ* may promote the conversion of Th17 cells into cells with a Th1-like phenotype (called Th17.1 cells) by virtue of the plasticity of Th17 cells. Th17.1 cells can produce IL-17 and IFN-*γ* simultaneously and express CCR6 and CXCR3; this expression is controlled by the transcription factors ROR*γ*t and T-bet. In many studies, Th17.1 cells are found to be pathogenic [188, 189]. A study on the EAE model shows that the development of Th17.1 cells requires T-bet and Runx1/3. T-bet deficiency or the inhibition of the transcriptional activity of Runx weakens the development of Th17.1 cells in EAE [190]. However, the molecular mechanisms that govern the generation of Th17.1 cells are unclear. At present, IFN-*γ* also seems to play an important role in the recurrence of uveitis [191], but the related mechanisms still need to be investigated.

### 11.3. Therapeutic Potential of Targeting IFN-*γ* in Autoimmune Uveitis

It has been reported that IFN-*γ* KO mice develop elevated Th17 cells and more severe local IL-17 responses and inflammation in the EAU model compared with WT counterparts, suggesting an inhibitory function of IFN-*γ* in Th17 cell differentiation [192]. Nevertheless, polarized IL-17-producing Th17 cells or IFN-*γ*-producing Th1 cells can drive the disease in recipients who lack mutual signal cytokines. EAU still develops in mice when IFN-*γ* or IL-17 is deficient [94]. Therefore, treating autoimmune uveitis with IFN-*γ* to inhibit the pathogenicity of Th17 cells may not be a suitable choice. Besides, a study found that treating EAU-susceptible B10.A mice with IFN-*γ* monoclonal antibodies increased the severity of EAU, while that with rIFN-*γ* ameliorated EAU. This suggests that EAU could be downregulated through the use of rIFN-*γ*, which may be due to the inhibition of Th17 cells [193]. However, a later report questioned the protective effect of IFN-*γ* in EAU because of the inhibitory effect of IFN-*γ* on myelopoiesis elicited by mycobacteria (from CFA) but lack of suppression of Th17 cell differentiation [194].

The levels of IFN-*γ* were elevated in the serum of patients with both active BD and VKH [195, 196]. Anti-IFN-*γ* has been reported to treat six cases of juvenile rheumatoid arthritis-associated uveitis. In four of the six patients, using anti-IFN-*γ* with standard treatment halved the duration and reduced the severity of the symptoms in the acute phase of the disease [197]. In addition, topical IFN-*γ* (IFN-*γ* 1b) was tested for treating cystoid macular edema (CME) secondary to uveitis, and it seemed to improve the CME (Clinicaltrials.gov NCT01376362). In brief, the treatment of uveitis with IFN-*γ* shows opportunities and challenges, while the drug targeting both Th1 and pathogenic Th17 cells or inducing them to transform into Tregs may be a better choice for treating autoimmune uveitis.

## 12. Conclusion

An increasing number of animal and clinical studies have shown the critical role of pathogenic Th17 cells in the initiation and progression of autoimmune uveitis. The mechanism of Th17 cell differentiation has been intensively studied in the past decade. In summary, IL-6, low concentration of TGF-*β*1, IL-1*β*, IL-23, and IL-21 have been proven to promote the differentiation of Th17 cells, while IL-27, IL-35, IL-2, IL-4, and IFN-*γ* can exert inhibitory effects. Several promising biological agents targeting these cytokines and their receptors have been developed. However, more details of the mechanisms need to be elucidated in the future due to the diversity of cytokine functions, the complicated microenvironments, and the plasticity of Th17 cells. Further investigations are needed to clarify the exact typical and pathogenic surface markers, transcription factors, and products of pathogenic Th17 cells in autoimmune uveitis and to find precise ways to induce the conversion of pathogenic cells to nonpathogenic phenotypes.

## Figures and Tables

**Table 1:** The clinical trials of agents target Th17 differentiation-associated cytokines.

Cytokine	Drug	Target	Dose	Autoimmune Uveitis	Reference or clinical trials
IL-6	Tocilizumab	IL-6R	4 or 8 mg/kg/i.v./every 4 weeks or 162 mg/s.c./every week	Refractory uveitis of BD, JIA-associated uveitis, NIU	[37], NCT03554161 [38] [39]
	Sarilumab	IL-6R	200 mg/s.c./every 2 weeks	Posterior segment NIU	[40]

IL-1*β*	Anakinra	IL-1*β* and IL-1*α*	100 mg/s.c./daily	BD-related uveitis	[76, 77]
	Canakinumab	IL-1*β*	150 mg/s.c./every 4, 6, or 8 weeks	BD-related uveitis	[76, 77]
	Gevokizumab	IL-1*β*	0.3 mg/kg/single; 30 or 60 mg/i.v. or s.c./every 4 weeks	BD-related uveitis, NIU	[78, 79], NCT01965145, NCT01684345, NCT01747538, NCT02375685, NCT02258854

IL-23	Ustekinumab	p40 subunit	90 mg/s.c/at week 1, week 4 and week 16; or 90 mg/s.c/every 4 weeks	BD-related uveitis, Active sight-threatening uveitis, noninfectious severe uveitis	NCT02648581 NCT02911116 NCT03847272

IL-2	Daclizumab	IL-2R*α*	2 mg/kg/s.c./every 2 weeks, twice, followed by 1 mg/kg/s.c./every 2 weeks; or 8 mg/kg/i.v./at week 1, 4 mg/kg/i.v./ at week 2, and 2 mg/kg/i.v. or s.c./every 4 weeks	NIU, JIA-associated uveitis, BD-related uveitis	[143, 146] [144] [145]
	Low-dose IL-2	—	1MUI/s.c./daily for five days, every week for 4 weeks, or 1MUI/s.c./daily for five days (day 1-day 5 in day 1-day 14) and then twice a week for 8 weeks	BD-related uveitis	NCT01988506, NCT04065672

IFN-*γ*	Anti-IFN-*γ*	IFN-*γ*	Not found	JRA-related uveitis	[197]
	IFN-*γ*1b	—	four drops (approximately 7 *μ*g per drop)/four times per day/1 week	CME secondary to uveitis	NCT01376362

NIU: noninfectious uveitis; i.v: intravenous; s.c: subcutaneous; IL-1Ra: IL-1R antagonist; 1MUI: one million units; CME: cystoid macular edema; JRA: juvenile rheumatoid arthritis.
